# CT-based radiomics nomogram for differentiating dedifferentiated liposarcoma from well-differentiated liposarcoma

**DOI:** 10.3389/fonc.2025.1683165

**Published:** 2025-10-07

**Authors:** Ting Yang, Ruo-Yu Chen, Yi-Fan Ding, Jing-Yan Wu, Ying Li, Jin-Wei Qiang

**Affiliations:** ^1^ Department of Radiology, Jinshan Hospital of Fudan University, Shanghai, China; ^2^ Department of Radiology, The First Affiliated Hospital of Soochow University, Suzhou, China

**Keywords:** retroperitoneal liposarcoma, radiomics, Ki-67, CT scan, nomogram

## Abstract

**Purpose:**

This study aimed to use radiomics features derived from plain CT scans to construct a model that can predict the pathological classification of Retroperitoneal liposarcoma (RLPS) preoperatively, help enhance preoperative planning and inform tailored treatment strategies.

**Methods:**

This retrospective study involving 114 consecutive RLPS patients from January 2022 to December 2024. Clinical, pathological, and CT imaging data were gathered. Radiomics features were extracted from plain CT scans and were selected through Least Absolute Shrinkage and Selection Operator (LASSO) regression. A radiomics signature was created, and a nomogram was developed for predicting dedifferentiated liposarcoma (DDLPS). Performance of the nomogram was assessed and compared with radiologist evaluation of the CT imaging. Area under the curve (AUC) and decision curve analysis in both training and validation sets.

**Results:**

Higher Ki-67 and unclear tumor boundary was established as an independent predictor for DDLPS. Five radiomics features were identified as significant predictors. a nomogram was developed by combining these features. The nomogram showed an AUC of 0.91 (95% CI: 0.84-0.98) and 0.89 (95% CI: 0.73-1.00) in the training and validation set, which outperforming the radiologist evaluation. Decision curve analysis confirmed that the nomogram provided a higher net clinical benefit compared to the radiologist.

**Conclusions:**

The radiomics nomogram significantly enhances the preoperative differentiation of RLPS subtypes.

## Introduction

Liposarcoma is the most common soft tissue sarcoma in adults, originating from primitive or embryonic lipoblasts (1). Approximately 10-15% of liposarcomas arise in the retroperitoneal space, known as retroperitoneal liposarcoma (RLPS), making it the most common primary retroperitoneal malignancy (2). RLPS can occur at any age, with a peak incidence in the 60s and 70s, showing no clear gender or ethnic predilection (3).

RLPS exhibits complex histological components and significant heterogeneity, which could be sub-categorized as well-differentiated liposarcoma (WDLPS) and dedifferentiated liposarcoma (DDLPS) (4). The primary treatment modality is radical surgical resection for RLPS. However, local recurrence post-surgery is frequent with poorer prognosis in DDLPS, which is the leading cause of death in RLPS patients (5). Thus, precise preoperative evaluation of the pathological subtype is vital for planning individualized treatment strategies (6).

Non-invasive imaging, including CT and MRI scans, is initially employed for diagnosing RLPS (7). However, they are difficult to accurately differentiate DDLPS and WDLPS. A recent meta-analysis on radiologist evaluation of RLPS showed that the diagnostic performance demonstrated summary sensitivity and specificity of only 0.85 and 0.63 for identifying DDLPS from WDLPS (8). Additionally, CT imaging may not identify the complex histological features of RLPS, limiting its ability to fully capture the tumor’s biological behavior (9).

Radiomics involves the extraction of numerous quantitative features from medical images to characterize tumor attributes, offering an objective method to assess the spatial heterogeneity of tumor tissues. A previous study reported that radiomic features could be used to identify G3 DDLPS from leiomyosarcoma at diagnosis (10). Recent studies also demonstrated the utility of radiomics in differentiating RLPS subtypes. For instance, Sudjai et al. found that radiomics analysis of MRI scans could distinguish these subtypes, where machine learning models surpassed traditional radiologist assessments (11). Although radiomics has gained traction for diagnosing and predicting the grading of various soft tissue tumors, its application to RLPS remains under-explored (12). These approaches are particularly effective in detecting dedifferentiated components, thereby potentially reducing reliance on invasive diagnostic procedures (13). Evidences also suggested that higher diagnostic accuracy is likely to be achieved through an integrated approach combining clinical and imaging scoring systems and/or radiomics (8). By integrating radiomics features with clinical risk factors, the radiomics nomogram could predict WDLPS from retroperitoneal lipomas preoperatively (14). However, to the best of our knowledge, there is few studies focused on radiomics feature analyses in differentiating DDLPS from WDLPS.

We hypothesize that radiomics features from plain CT scans can accurately predict the histopathological classification of RLPS. Thus, this study aims to develop a predictive model enabling differentiation between DDLPS and WDLPS utilizing radiomics features derived from plain CT scans, and to enhance preoperative planning and tailor treatment strategies.

## Materials and methods

### Ethnics

This study was conducted following the principles outlined in the Declaration of Helsinki and was reviewed and approved by the Ethics Committee of Jinshan Hospital of Fudan University (JIEC2025S01). And the patient informed consent was waived due to the retrospective nature of the study, All the information of patients is anonymized and replaced by numerical numbers.

### Data collection

From January 2022 to December 2024, a retrospective collection of clinical, pathological, and imaging data was conducted from consecutive RLPS patients who underwent surgical treatment at local hospital. The inclusion criteria for this study were (1): postoperative pathology confirmed RLPS (2); CT examination within 30 days before surgery (3); no history of other tumors and metastatic diseases. Exclusion criteria were (1): unclear pathological diagnosis (2); poor CT image quality, missing or incomplete data. Ultimately, 114 cases of RLPS were included, with 44 cases of WDLPS and 70 cases of DDLPS. The patients were randomly divided into training set and validation set according to a ratio of 7:3.

Clinical and pathological data include age, gender, marker of proliferation Ki-67 (Ki-67), cyclin-dependent kinase 4 (CDK4) and mouse double minute 2 homolog (MDM2) were retrieved from the electrical records.

### CT scanning

This study used a spiral CT (Aquilion ONE TSX-301C, Canon, Japan) for abdominal examinations. Patients were in a supine position, head first, with arms raised, aligned to the mid-axillary line, and scanned after holding their breath post-inhalation. The scanning range was from the diaphragm to the pubic symphysis. Scanning parameters were: tube voltage 120 kV, tube current 242 mA, collimation width 16×1.2 mm, pitch 1.0625, scanning time 5–7 s, field of view (FOV) 400 mm×400 mm, and image matrix 512×512. Thin-layer reconstruction was performed using a soft tissue reconstruction algorithm sequence, with a reconstruction layer thickness and interval of 1 mm.

### CT imaging evaluation and tumor segmentation

The CT imaging was fist reviewed by two radiologists (with 3 and 15 years of experience in abdominal imaging). Three CT features were evaluated (15): tumor margin (clear or unclear), tumor density (fat-like or mixed with solid masses), and satellite nodules (positive/negative focal nodular density area). The cases showed any of unclear tumor margin, mixed tumor density or positive satellite nodules were considered as DDLPS. In cases of significant discrepancies, the observations were reviewed by a more experienced radiologist (with 35 years of experience in abdominal imaging). All the radiologists were blinded to the clinical and pathological results.

Then, regions of interest (ROIs) of all cases were delineated using ITK-SNAP software. CT images were imported into the software, adjusted to the soft tissue window, and manually delineated based on axial images along each tumor layer, avoiding structures like blood vessels and abdominal organs to generate the volume of interest (VOI). This process was manually completed by one radiologist. Selecting the most prominent lesion as the main subject for delineation and preferably choosing the tumor’s central layer, avoiding adjacent organs and artifacts were payed attention to reduce potential bias. To assess reproducibility, 30 patients were randomly selected for ROI delineation by the same radiologist and another radiologist one month later. Interclass and intraclass correlation coefficients (ICCs) were calculated to evaluate the consistency of radiomic feature measurements.

### Radiomics data processing and feature extraction and selection

Radiomics features of the delineated VOIs were extracted and screened using Pyradiomics (https://pypi.org/project/pyradiomics/) following the Imaging Biomarker Standardization Initiative (IBSI) standards (https://arxiv.org/abs/1612.07003). After normalizing all images, first-order, second-order, and high-order features were extracted from the VOI images. Features with ICCs less than 0.75 were considered unstable and removed. Then features with Pearson correlation coefficients greater than 0.9 were considered as redundant features and eliminated. The remaining features were further screened using the Least Absolute Shrinkage and Selection Operator (LASSO) with 10-fold cross-validation to obtain non-zero coefficient radiomic features. These optimal features were linearly fitted to generate radiomics scores (radscore) for each cases based on their respective weighted coefficients.

### Radiomics Nomogram Construction

Based on the optimal radiomic features of the training set, a radiomic model (radscore) was constructed to predict tumor pathological subtypes of RLPS. Patients’ clinical data and radiologist-assessed imaging features were selected as clinical features using multivariate logistic regression as a clinical model. A radiomics nomogram was constructed by combining the radscore and selected clinical features for predicting DDLPS.

### Discrimination of the radiomics model, clinical model, radiologist evaluation and radiomics nomogram

The radiomics model, clinical model, radiologist evaluation and radiomics nomogram were validated. The area under the receiver operating characteristic (ROC) curves (AUC) were calculated to assess models performance. Calibration curves were drawn after the Hosmer-Lemeshow test to evaluate the fit and predictive performance of the radiomics nomogram in both the training and validation sets.

### Clinical application value

To assess the clinical applicability of radiomics nomogram, a clinical decision curve was used to compare the net clinical benefits of RLPS patients based on the result of radiologist evaluation and radiomics nomogram at different probability thresholds.

### Statistical analysis

Statistical analyses were conducted using R software (4.4.2, https://www.r-project.org/). Descriptive statistics for quantitative data were expressed as mean ± standard deviation (SD). The Shapiro-Wilk test was used to test for normal distribution. Independent sample t-test compared clinical indicators between the WDLPS and DDLPS groups. Qualitative data comparisons of clinical and CT feature used chi-square or Fisher’s exact test. DeLong test for evaluating the ROC curve performance between different models. A p-value < 0.05 was considered statistically significant.

## Results

### Clinical characteristics

**Figure 1:**
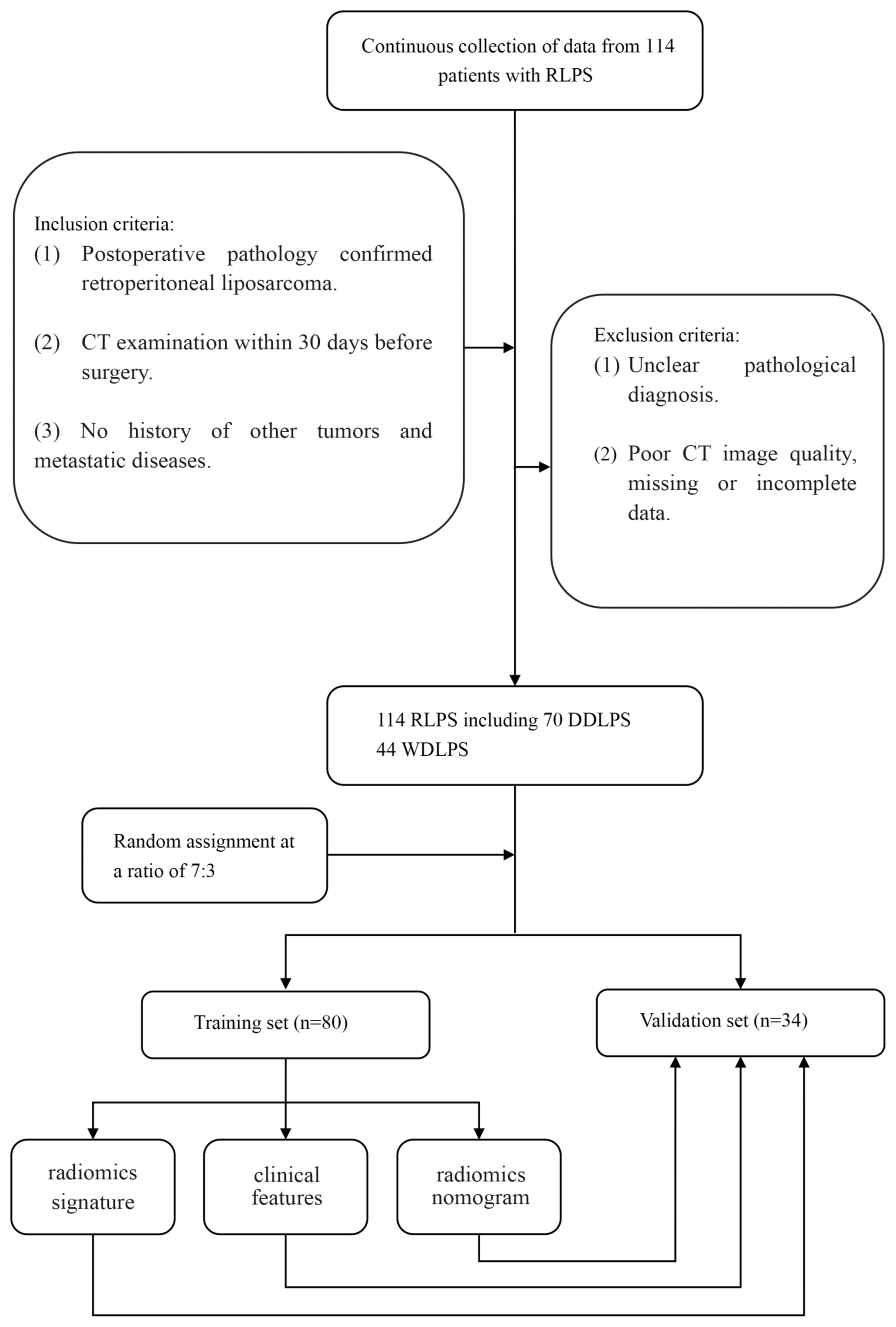
The work flow of this study.

There were 80 cases in the training set, including 31 cases of WDLPS, 49 cases of DDLPS, and 34 cases in the validation set, including 13 cases of WDLPS and 21 cases of DDLPS. There were no significant differences in age, gender, CDK4, MDM2 and satellite nodules in tumors between WDLPS and DDLPS in both training and validation sets, while the KI-67, tumor margin and tumor density were statistically significant in both training and validation sets. The clinical characteristics of the training and validation sets are shown in **Table 1**. The work flow of this study is shown in **Figure 1**.

**Table 1:** The clinical characteristics of the training and validation set.

Parameters	Training set		P-value	Verification set		P-value
	WDLPS (N = 29)	DDLPS (N = 51)		WDLPS (N = 15)	DDLPS (N = 19)	
Radscore	0.440 (0.185)	0.721 (0.242)	<0.001	0.394 (0.309)	0.756 (0.188)	0.001
Gender						
Female	10 (32.3%)	21 (42.9%)	0.476	5 (38.5%)	10 (47.6%)	0.867
Male	21 (67.7%)	28 (57.1%)		8 (61.5%)	11 (52.4%)	
Age	60.0 (11.1)	56.3 (10.9)	0.148	54.8 (11.0)	60.8 (8.41)	0.106
KI67	0.113 (0.104)	0.211 (0.125)	<0.001	0.0831 (0.101)	0.105 (0.0854)	0.051
CDK4						
Negative	1 (3.2%)	1 (2.0%)	1.000	13 (100%)	20 (95.2%)	1
Positive	30 (96.8%)	48 (98.0%)		0 (0%)	1 (4.8%)	
MDM2						
Negative	2 (6.5%)	7 (14.3%)	0.473	13 (100%)	16 (76.2%)	0.16
Positive	29 (93.5%)	42 (85.7%)		0 (0%)	5 (23.8%)	
Tumor margin						
Clear	21 (67.7%)	8 (16.3%)	<0.001	7 (53.8%)	2 (9.5%)	0.014
Unclear	10 (32.3%)	41 (83.7%)		6 (46.2%)	19 (90.5%)	
Tumor density						
Fat-like	23 (74.2%)	13 (26.5%)	<0.001	9 (69.2%)	4 (19.0%)	0.01
Mixed	8 (25.8%)	36 (73.5%)		4 (30.8%)	17 (81.0%)	
Satellite nodules						
Negative	23 (74.2%)	29 (59.2%)	0.258	13 (100%)	12 (57.1%)	0.019
Positive	8 (25.8%)	20 (40.8%)		0 (0%)	9 (42.9%)	
Radiologists						
DDLPS	12 (38.7%)	43 (87.8%)	<0.001	6 (46.2%)	20 (95.2%)	0.004
WDLPS	19 (61.3%)	6 (12.2%)		7 (53.8%)	1 (4.8%)	

DDLPS, dedifferentiated liposarcoma; MDM2, Mouse Double Minute 2; WDLPS, well-differentiated liposarcoma

### CT imaging evaluation

**Figure 2:**
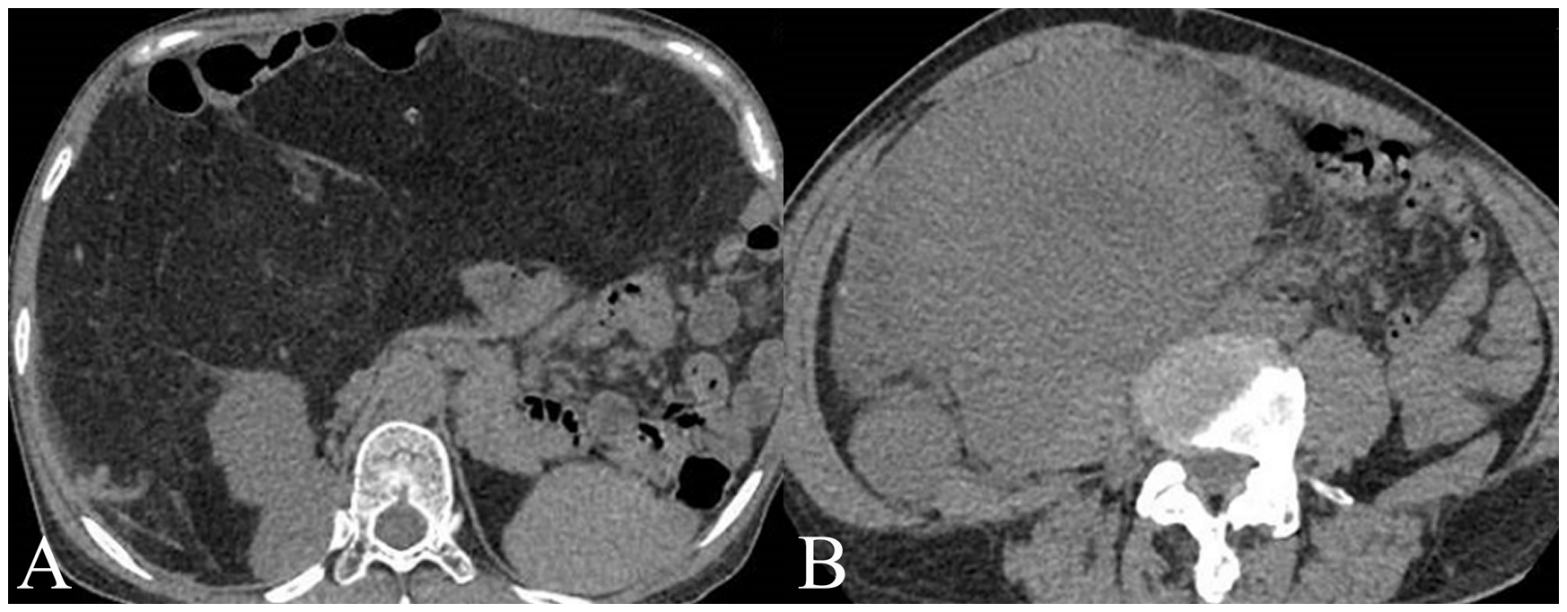
Two cases examples of histology confirmed well-differentiated liposarcoma (WDLPS) and dedifferentiated liposarcoma (DDLPS). **(A)** A WDLPS located in the upper abdomen, has clear boundaries with fat-like content, pushing the surrounding tissues. **(B)** A DDPLS located in the right lower abdomen, with unclear separations from surrounding tissues with solid content invading the right psoas major muscle.

For the training set, among WDLPS cases, radiologists classified 19 cases (61.3%) correctly as WDLPS and 12 cases (38.7%) incorrectly as DDLPS. For DDLPS cases, radiologists correctly identified 43 cases (87.8%) as DDLPS, while 6 cases (12.2%) were incorrectly classified as WDLPS. For the validation set, among WDLPS cases, radiologists classified 7 cases (53.8%) correctly as WDLPS and 6 cases (46.2%) incorrectly as DDLPS. For DDLPS cases, radiologists correctly identified 20 cases (95.2%) as DDLPS, while 1 cases (4.8%) were incorrectly classified as WDLPS. Two cases demonstration is shown in **Figure 2**.

### Radiomics features selection

**Figure 3:**
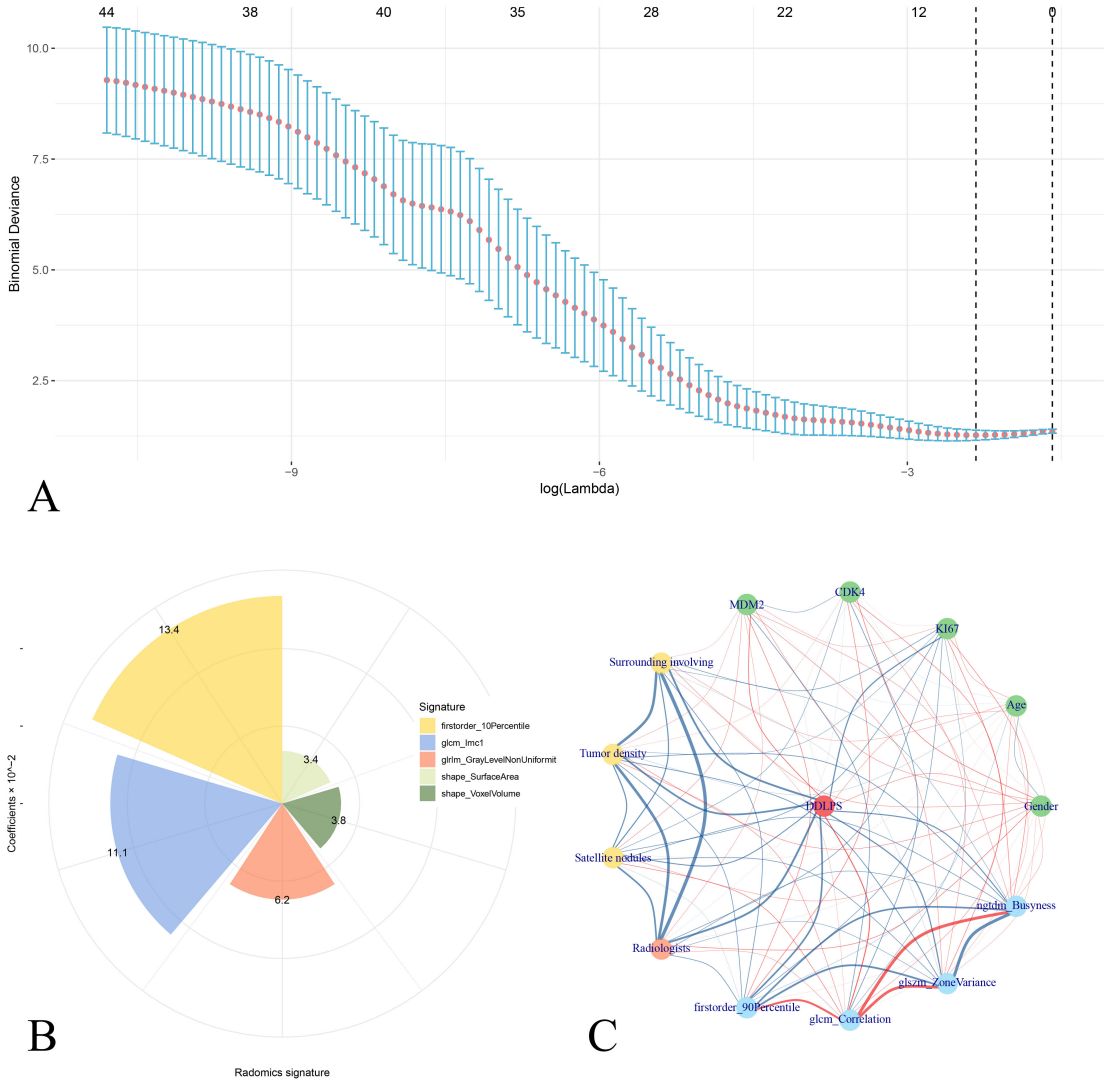
Analysis of radiomics signatures and correlation interactions with clinical features. **(A)** LASSO regression analysis showing binomial deviance across different log(lambda) values, with error bars representing confidence intervals. Five radiomics features is selected as the radiomics signatures. **(B)** Pie chart shows the distribution of radiomics signatures with their coefficients in liner combining. **(C)** Correlation network of radiomics signatures and clinical features for DDLPS illustrates the relationships between clinical variables (green nodes), radiomics signatures (blue nodes), and the DDLPS outcome (red central node). Red edges represent positive correlations, while blue edges indicate negative correlations. The thickness of each edge corresponds to the strength of the correlation.

A total of 107 radiomics features were extracted, and 5 optimal radiomics features were selected. The radscore was calculated as Radscore = 0.76755 + 0.03428×shape_SurfaceArea+0.03798×shape_VoxelVolume+0.13445×firstorder_10Percentile+0.11145×glcm_Imc1 + 0.06222×glrlm_GrayLevelNonUniformity. The corelations of radiomics features and the clinical features is shown in **Figure 3**.

### Radiomics nomogram construction

**Figure 4:**
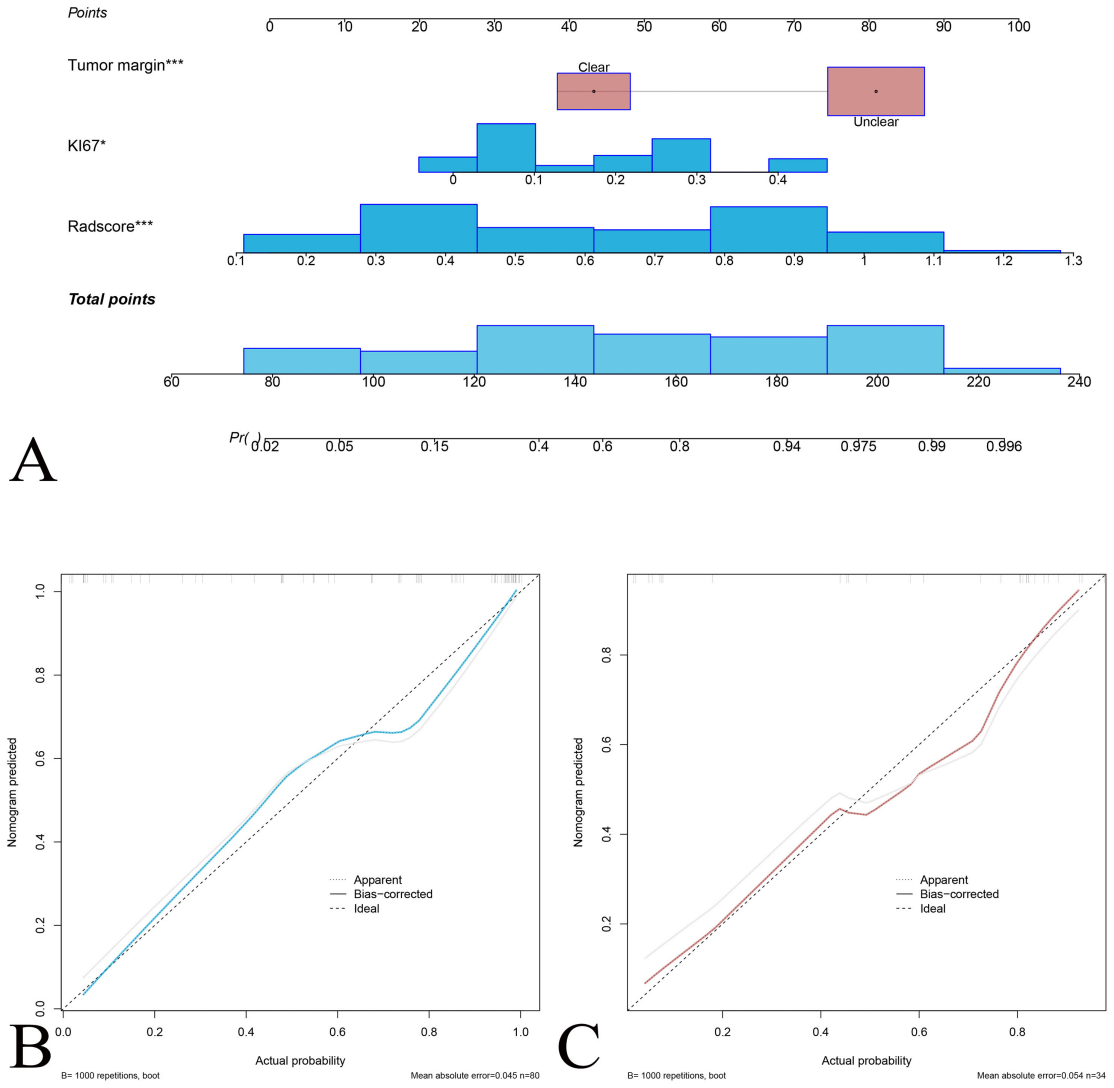
Nomogram for predicting the probability of DDLPS recurrence. **(A)** The nomogram integrates tumor margin, Ki-67 expression, and radscore, and total points to estimate the DDLPS probability. Each variable is assigned a score on the “Points” scale, and the total score is mapped to a probability of DDLPS. Calibration curve for the nomogram prediction model for the training set **(B)** and the validation set **(C)**. The solid blue (red) line represents the agreement between predicted and observed probabilities, the dashed line represents perfect prediction (ideal), and the gray line indicates bias-corrected predictions.

The radiomics model (radscore) was constructed to predict the pathological classification of DDLPS. Multivariate logistic regression analysis showed that Ki-67 and tumor margin were clinical independent risk factors for predicting DDLPS (they were liner fitted as a clinical model). The radiomics nomogram was constructed combined the radscore with clinical independent risk factors to predict DDLPS (**Figure 4**). Calibration curves showed good agreement between predicted and observed results of the radiomics nomogram in both training and validation sets.

Discrimination of the Radiomics Model, Clinical Model, Radiologist Evaluation and Radiomics Nomogram(**Table 2**).

**Table 2:** Discrimination performance of radiomics model, clinical model, radiologist’s evaluation, and radiomics nomogram.

Data sets	Models	AUC	95%CI	SPE	SEN	NPV	PPV	P*
Training set	Radscore	0.81	0.71-0.91	0.81	0.73	0.66	0.86	0.019
	Clinical model	0.83	0.74-0.93	0.68	0.88	0.78	0.81	0.023
	Radiologists	0.75	0.65-0.84	0.61	0.88	0.76	0.78	<0.001
	Nomogram	0.91	0.84-0.98	0.87	0.88	0.82	0.91	–
Validation set	Radscore	0.84	0.67-1.00	0.85	0.76	0.69	0.89	0.020
	Clinical model	0.79	0.64-0.95	0.62	0.90	0.80	0.79	0.193
	Radiologists	0.75	0.60-0.89	0.54	0.95	0.87	0.77	0.013
	Nomogram	0.89	0.73-1.00	0.92	0.90	0.86	0.95	–

*Compared with nomogram; AUC, area under the curve; CI, confidence interval; SPE specificity; SEN, sensitivity; NPV, negative predictive value; PPV, positive predictive value.

AS showed in **Table 2**, in the training set, the AUC of the radiomics model was 0.81 (95%CI: 0.71-0.91) with sensitivity and specificity of 0.73 and 0.81. In the validation set, the AUC of the radiomics model was 0.84 (95%CI: 0.67-1.00) with sensitivity and specificity of 0.76 and 0.85.

In the training set, the AUC of the clinical model was 0.83 (95%CI: 0.74-0.93) with sensitivity and specificity of 0.88 and 0.68. In the validation set, the AUC of clinical model was 0.79 (95%CI: 0.64-0.95) with sensitivity and specificity of 0.90 and 0.62.

In the training set, the AUC of the radiologist evaluation was 0.75 (95%CI: 0.65-0.84) with sensitivity and specificity of 0.88 and 0.61. In the validation set, the AUC of the radiomics nomogram was 0.75 (95%CI: 0.60-0.89) with sensitivity and specificity of 0.95 and 0.54.

In the training set, the AUC of the radiomics nomogram was 0.91 (95%CI: 0.84-0.98) with sensitivity and specificity of 0.87 and 0.88. In the validation set, the AUC of the radiomics nomogram was 0.89 (95%CI: 0.73-1.00) with sensitivity and specificity of 0.90 and 0.92.

The DeLong test showed better discrimination performance of radiomics nomogram than the radiologist evaluation both in training and validation sets (both P<0.001).

### Clinical application value

**Figure 5:**
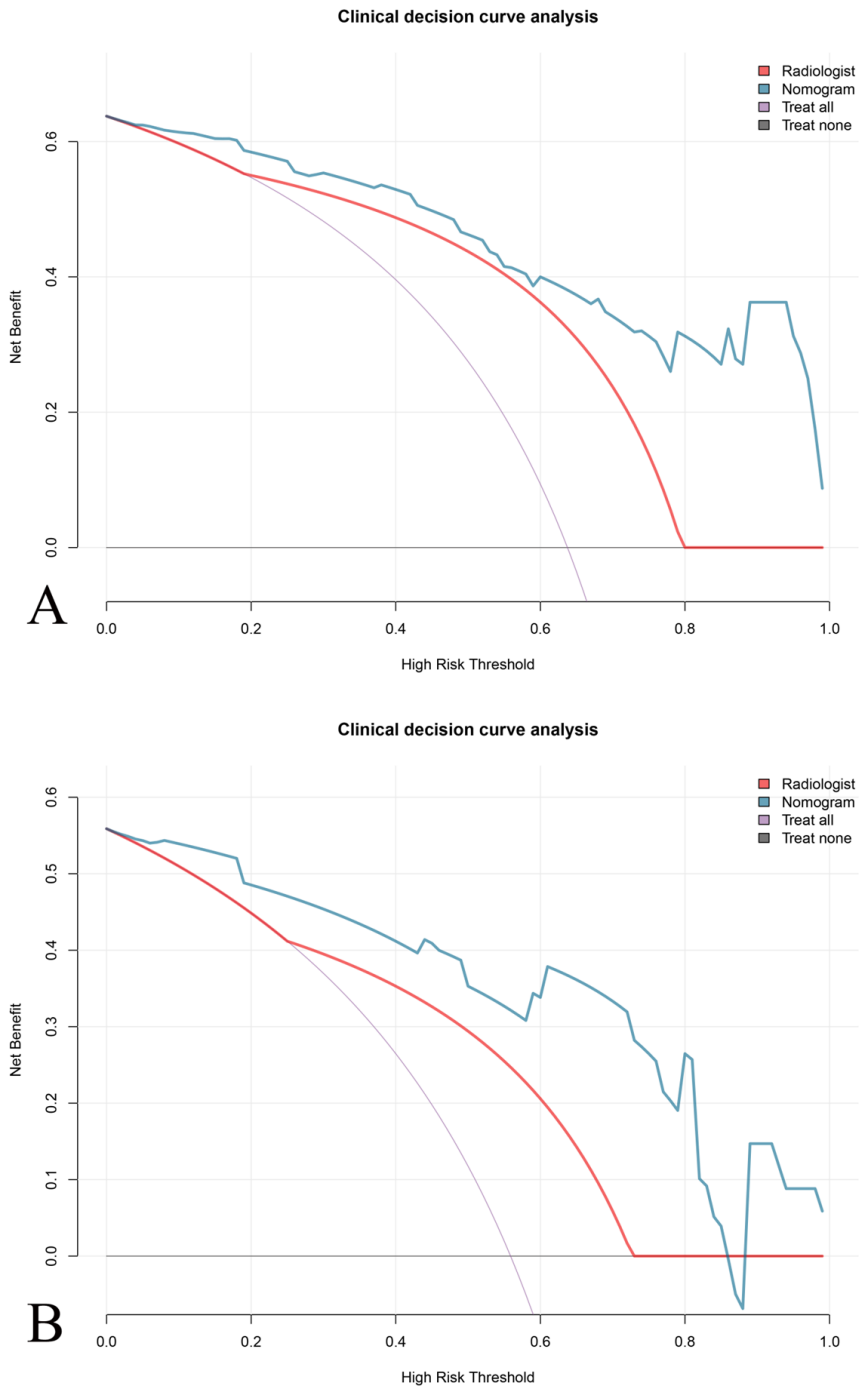
Clinical decision curve analysis (DCA) for evaluating the net benefit of different prediction models. DCA comparing the predictive performance of the \the nomogram (blue line), the radiologist evaluation (red line), and the “treat all as DDLPS” (purple line) and “treat none as DDLPS” (gray line) strategies. The nomogram demonstrates a higher net benefit across a range of thresholds compared to the radiologist evaluation in both **(A)** training and **(B)** validation set.

The clinical decision curve showed that under a certain probability threshold, both the radiomics feature and the imaging nomogram could effectively predict the pathological classification of DDLPS and improve the clinical net benefit of patients compared with the treatment of “total intervention” or “zero intervention”. Moreover, under most probability thresholds, decision making based on the imaging nomogram results in a higher clinical net benefit for patients compared to the radiologist evaluation (**Figure 5**).

## Discussion

This study developed a radiomics nomogram combining features from plain CT scans (tumor margin), Ki-67 expression and five significant radiomic features to differentiate between WDLPS and DDLPS preoperatively. The nomogram demonstrated strong performance, which was better than standalone of radiologist evaluation. Decision curve analysis confirmed that the nomogram significantly improved the net clinical benefit across various probability thresholds. The study highlights the potential of combining radiomics with clinical biomarkers to enhance preoperative diagnosis and aid in treatment planning for patients with RLPS.

Preoperative evaluation of the pathological classification of RLPS is crucial for selecting appropriate treatment strategies and significantly influences prognosis and recurrence risk. In this study, we identified Ki-67 expression as an independent clinical predictor for distinguishing between WDLPS and DDLPS, which is in accordance with previous studies (6). Ki-67, a well-established proliferation marker in clinical practice (16), which is emphasized in prior studies of DDLPS (17). Preoperative sampling for Ki-67 expression may complement the assessment of tumor heterogeneity. Moreover, integrating Ki-67 into the radiomics model emphasizes the importance of using both biological and imaging markers to refine diagnostic accuracy.

Tumor margin is found important in differentiating DDLPS from WDLPS. As previous study reported that WDLPS typically presents with well-defined, clear margins, while DDLPS often shows irregular or poorly defined borders due to the presence of more aggressive and infiltrative areas (1). Tumor density also provides valuable insights, as WDLPS tends to exhibit a homogeneous fat-like density, characteristic of its well-differentiated adipose tissue composition (18). In contrast, DDLPS is more likely to present with mixed densities, including areas of solid masses interspersed with fat, reflecting its more aggressive nature and the presence of non-fatty, dedifferentiated tissue (18). Other study reported that DDLPS often showing positive satellite nodules due to its tendency to invade surrounding tissues, whereas WDLPS typically does not present with these nodular foci (3). Satellite nodules was not found difference between WDLPS and DDLPS, which might be because non-contrasted CT was used in this study and satellite nodules would be more clearly visible under contrasted CT. However, our study’s reliance on plain CT imaging highlights the accessibility and cost-effectiveness of this approach. While contrast-enhanced CT are commonly used in soft tissue sarcoma diagnosis, plain CT scans are widely available and circumvent the risks associated with contrast agents, such as allergic reactions or nephrotoxicity. This makes our radiomics nomogram particularly beneficial in resource-limited settings or for patients with contraindications to contrast agents.

The study underscores the utility of radiomics in identifying subtle heterogeneity within RLPS tumors, with the selected radiomics features providing insights into tumor size (Shape_VoxelVolume), growth patterns (Shape_SurfaceArea), intensity (Firstorder_10Percentile, indicate areas of fat, solid, necrosis or cystic within tumors), and tumor heterogeneity (GLCM_Imc1 and GLRLM_GrayLevelNonUniformity), which are critical for identifying dedifferentiated regions. For instance, Shape_SurfaceArea and Shape_VoxelVolume reflect tumor size and growth patterns, which are directly associated with aggressive infiltration in DDLPS compared to the more circumscribed WDLPS. Firstorder_10Percentile is an intensity-based feature, which captures low-attenuation regions that may correspond to necrotic, cystic, or fatty components, aligning with the heterogeneous tissue composition of DDLPS. GLCM_Imc1 reflects local intensity variations and structural complexity, consistent with the disorganized cellular and stromal architecture of DDLPS. GLRLM_GrayLevelNonUniformity mirrors the biological diversity of tumor microenvironments in dedifferentiated regions. These results extend and align with recent research on the role of radiomics in classifying RLPS. The use of radiomics allows for the identification of these subtle features that may not be fully appreciated through traditional visual assessment, thus improving diagnostic accuracy. Several other studies have explored the use of CT radiomics in identifying DDLPS and WDLPS, with similar aims but differing methodologies and findings. One study emphasized the challenges in distinguishing DDLPS from WDLPS by CT based radiomics, highlighting the difficulty in visualizing the complex heterogeneity of these tumors. They showed that radiomics model can predict the histological type and grade of RLPS with excellent performance (12). Moreover, studies have explored radiomics for soft tissue tumors, including liposarcomas, using CT scans, and reported AUC values of over 0.80 for distinguishing subtypes (10, 19).

Although CT findings such as irregular tumor margins, mixed densities, and satellite nodules are not exclusive to DDLPS and may occasionally be seen in WDLPS, prior studies have shown these features to be significantly more common in dedifferentiated tumors. WDLPS typically presents as a homogeneous, fat-like mass with well-circumscribed borders, whereas DDLPS often demonstrates infiltrative, irregular margins and heterogeneous densities due to the presence of non-adipose, dedifferentiated tissue (8, 15). Similarly, focal nodular non-adipose areas have been associated with aggressive histologic transformation, though their detection is more reliable on contrast-enhanced CT (3). In our cohort, margin and density differences reached statistical significance between WDLPS and DDLPS groups, supporting their inclusion. Importantly, these qualitative features were not used in isolation but were combined with Ki-67 expression and radiomics-derived heterogeneity markers, resulting in a predictive model that outperformed subjective radiologist assessment. This integrative approach underscores the added value of radiomics in extracting subtle imaging signatures beyond conventional CT interpretation (12).

Furthermore, The findings suggest that integrating radiomics data with clinical biomarkers presents a promising approach for personalized treatment planning in RLPS patients, supporting the shift towards precision oncology (20). The combination of Ki-67, CT features and radiomics features is notably significant, bridging the gap between imaging and molecular pathology. This synergy allows for a more comprehensive understanding of tumor biology, thereby improving diagnostic precision. This aligns with the principles of precision medicine, where treatment decisions are informed by multimodal data (21). The nomogram could aid in preoperative planning by identifying patients with likely DDLPS who may benefit from more extensive surgical resection, closer follow-up, or consideration for neoadjuvant therapies. These findings reinforce the consensus that radiomics, when integrated with clinical biomarkers, is a potent tool for enhancing the precision of RLPS diagnosis and treatment planning (22). Furthermore, our model relies only on plain CT scans, it is particularly suitable for resource-limited settings or patients contraindicated for contrast-enhanced imaging.

However, this study has several limitations. Firstly, its retrospective nature and small sample size might affect the generalizability of the results. Future research should aim to validate these models in larger, multicenter cohorts and investigate their potential in predicting therapeutic responses and long-term outcomes. Second, the model was exclusively developed for differentiating between WDLPS and DDLPS, not accounting for other specific pathological subtypes. Lastly, only plain CT images were used in this study, contrast-enhanced imaging could provide complementary information not captured in this study. Future studies might benefit from incorporating enhanced CT imaging with an expanded data-set.

## Conclusions

In conclusion, the integration of radiomics features from plain CT scans with Ki-67 expression in a nomogram significantly enhances the preoperative differentiation of RLPS subtypes. This approach could lead to more precise treatment planning, potentially improving patient outcomes. Further validation in larger, multi-center studies is necessary to confirm these findings and extend their applicability.

## Data Availability

The raw data supporting the conclusions of this article will be made available by the authors, without undue reservation.
